# The buccohypophyseal canal is an ancestral vertebrate trait maintained by modulation in sonic hedgehog signaling

**DOI:** 10.1186/1741-7007-11-27

**Published:** 2013-03-28

**Authors:** Roman H Khonsari, Maisa Seppala, Alan Pradel, Hugo Dutel, Gaël Clément, Oleg Lebedev, Sarah Ghafoor, Michaela Rothova, Abigael Tucker, John G Maisey, Chen-Ming Fan, Maiko Kawasaki, Atsushi Ohazama, Paul Tafforeau, Brunella Franco, Jill Helms, Courtney J Haycraft, Albert David, Philippe Janvier, Martyn T Cobourne, Paul T Sharpe

**Affiliations:** 1 Department of Craniofacial Development and Stem Cell Research, Comprehensive Biomedical Research Center, Dental Institute, King’s College London, London, UK; 2 Service de Chirurgie Maxillo-Faciale, Centre Hospitalier Universitaire Hôtel-Dieu, Nantes, France; 3 Department of Orthodontics, Dental Institute, King’s College London, Guy’s Hospital, London, UK; 4 American Museum of Natural History, New York City, NY, USA; 5 CNRS-UMR 7207, Muséum National d’Histoire Naturelle, Paris, France; 6 CNRS-UMR 7179, Muséum National d’Histoire Naturelle, Paris, France; 7 Paleontological Institute of Russian Academy of Science, Moscow, Russian Federation; 8 Institute of Experimental Medicine, Academy of Sciences of the Czech Republic, Prague, Czech Republic; 9 Department of Embryology, Carnegie Institution of Washington, Baltimore, USA; 10 European Synchrotron Radiation Facility, Grenoble, France; 11 Department of Pediatrics, Università degli Studi di Napoli Federico II, Naples, Italy; 12 Department of Surgery, Stanford University, Palo Alto, CA, USA; 13 College of Dental Medicine, Craniofacial Biology, Medical University of South Carolina, Carolina, SC, USA; 14 Service de Génétique Clinique, Centre Hospitalier Universitaire Hôtel-Dieu, Nantes, France

**Keywords:** Buccohypophyseal canal, Chondrichthyans, Coelacanth, Endoderm, Knock-out mouse, Midline, Neural crest, Primary cilia, Shh

## Abstract

**Background:**

The pituitary gland is formed by the juxtaposition of two tissues: neuroectoderm arising from the basal diencephalon, and oral epithelium, which invaginates towards the central nervous system from the roof of the mouth. The oral invagination that reaches the brain from the mouth is referred to as Rathke’s pouch, with the tip forming the adenohypophysis and the stalk disappearing after the earliest stages of development. In tetrapods, formation of the cranial base establishes a definitive barrier between the pituitary and oral cavity; however, numerous extinct and extant vertebrate species retain an open buccohypophyseal canal in adulthood, a vestige of the stalk of Rathke’s pouch. Little is currently known about the formation and function of this structure. Here we have investigated molecular mechanisms driving the formation of the buccohypophyseal canal and their evolutionary significance.

**Results:**

We show that Rathke’s pouch is located at a boundary region delineated by endoderm, neural crest-derived oral mesenchyme and the anterior limit of the notochord, using *CD1*, *R26R-Sox17-Cre* and *R26R-Wnt1-Cre* mouse lines. As revealed by synchrotron X-ray microtomography after iodine staining in mouse embryos, the pouch has a lobulated three-dimensional structure that embraces the descending diencephalon during pituitary formation. *Polaris*^*fl/fl*^*; Wnt1-Cre*, *Ofd1*^*-/-*^ and *Kif3a*^*-/-*^ primary cilia mouse mutants have abnormal sonic hedgehog (Shh) signaling and all present with malformations of the anterior pituitary gland and midline structures of the anterior cranial base. Changes in the expressions of *Shh* downstream genes are confirmed in *Gas1*^*-/-*^ mice. From an evolutionary perspective, persistence of the buccohypophyseal canal is a basal character for all vertebrates and its maintenance in several groups is related to a specific morphology of the midline that can be related to modulation in Shh signaling.

**Conclusion:**

These results provide insight into a poorly understood ancestral vertebrate structure. It appears that the opening of the buccohypophyseal canal depends upon Shh signaling and that modulation in this pathway most probably accounts for its persistence in phylogeny.

## Background

The buccohypophyseal canal (BHC) is a peculiar duct connecting the floor of the diencephalon and the adenohypophysis with the roof of the mouth. In all mammals, it closes in the early stages of development (embryonic day (E) 11.5 in CD1 mice). Its persistence in humans represents an abnormality that occurs in a wide range of disorders of the midline, such as holoprosencephaly [1]. An open canal is in fact very rare in living adult gnathostomes (jawed vertebrates) and entirely lacking in adult tetrapods: within this group, the BHC only persists in some larval lungfishes [2] and lissamphibians [3]. By contrast, an open BHC is very common in most fossil early gnathostomes, in particular Paleozoic fishes [2]. Surprisingly, even though a connection between the mouth and the central nervous system may play some physiological role, the mode of formation and functions of the BHC are poorly understood.

The BHC is a vestige of Rathke’s pouch (RP), which is formed from the ectodermal diverticulum of the adenohypophysis after its separation from the roof of the mouth. RP derives from the hypophyseal placode, which is initially an epithelial thickening in a small oral region underlying the diencephalon, where expression of the midline signaling molecule sonic hedgehog (Shh) is down-regulated. However, Shh target genes are expressed throughout the walls of the RP and are required for its normal outgrowth and differentiation [4]. In mice, the pouch starts forming around E9.5 and the transient BHC is ultimately closed by E11.5. In species where a persistent BHC is present at adult stages, and in mutants with an open BHC, the pouch does not close and foramina are maintained on the intracranial and/or oral sides of the basisphenoid bone, sometimes coalescing with foramina of the internal carotid arteries [5].

RP is located at the rostral end of the notochord [6] at the foremost limit of the gut endoderm [7] and marks the posterior limit of the neural crest-derived territories in the skull base [8,9]. Its position at three important tissue boundaries [10] exposes this region to numerous signaling pathways involved in cranial base morphogenesis, such as Shh [11]. Shh is in fact secreted both by the rostral tip of the notochord [11,12] and the most rostral zone of the foregut endoderm [13]. While the positions of the RP relative to the rostral end of the notochord and to the posterior border of the neural crest-derived tissues are well documented, there prevails an uncertainty about its position in relation to the gut endoderm [14,15].

In this study, we first provide definitive proof of the relative position of endodermal cells to the RP by using *R26R-Sox17-Cre* reporter mice, and thus underline the high exposure of this anatomical region to morphogens involved in midline formation. In particular, knowing the important role of Shh in facial midline development [16,17] and pituitary formation [4], we also investigated persistence of the BHC in four Shh pathway-related transgenic mouse lines: *Polaris*^*fl/fl*^*;Wnt1-Cre*, *Ofd1*^*-/-*^*Kif3a*^*-/-*^ and *Gas1*^*-/-*^. The expression changes of Shh downstream genes were investigated in *Gas1*^*-/-*^ mice using *in situ* hybridization. Finally, we performed a comparative study of species that retain an open canal in order to describe the distribution of BHC persistence amongst vertebrates and we have attempted to relate the presence or absence of the BHC with modifications in midline anatomy that could be due to modulation in Shh signaling.

## Results

### The buccohypophyseal canal derives from an ectodermal placode located close to three developmental boundaries

In the absence of conclusive data, we first aimed to confirm that RP is located at the anterior limit of the oral endoderm in the roof of the oral cavity by using *R26R-Sox17-Cre* mice. *Sox17* is a reliable early endoderm marker essential for its differentiation [18].

**Figure 1:**
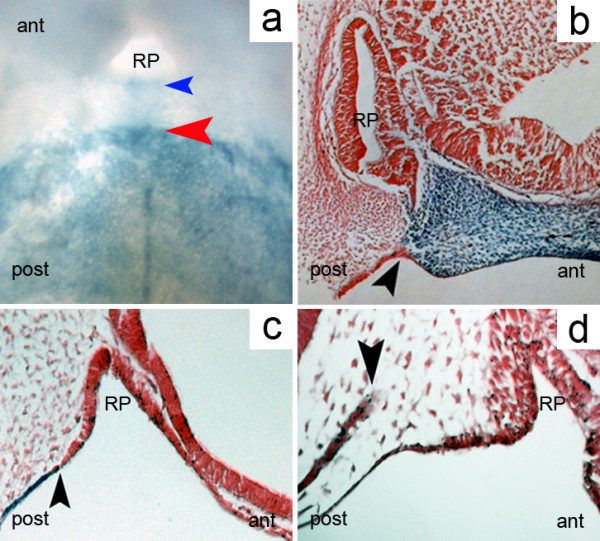
**Rathke’s pouch is located at a triple boundary. (a)** Whole-mount LacZ staining of an *R26R-Sox17-Cre* E10.5 mouse embryo showing the anterior limit of the endoderm (red arrowhead) and the posterior border of Rathke’s pouch (blue arrowhead); endobuccal view of the oral roof. **(b)** Anterior limit of the endoderm in the mid-sagittal plane at E10.5 (arrowhead), LacZ staining, *R26R-Sox17-Cre* mouse embryo. **(c)** Posterior limit (arrowhead) of the neural crest-derived mesoderm in the mid-sagittal plane at E12.5, corresponding to the location of the closing buccohypophyseal canal, in an *R26R-Wnt1-Cre* mouse embryo. **(d)** Anterior end of the notochord (arrowhead), eosin staining, E10.5, wild-type mouse embryo ant, anterior; post; posterior; RP, Rathke’s pouch.

*Sox17*-positive cells extended to the border of the invaginating RP and also to some rare endoderm cells populating a region corresponding to the former localization of the buccopharyngeal membrane. However, there was no contribution of endoderm to RP (Figure 1a,b). Following this, we used *R26R-Wnt1-Cre* reporter mice to confirm previously published data that RP is bordered anteriorly by neural crest cells (Figure 1c). We have also illustrated the position of the tip of the notochord behind the posterior wall of the pouch (Figure 1d). Together, these findings confirm the position of RP at the intersection of three important boundaries, where molecules involved in midline formation intersect [16,17,19,20].

**Figure 2:**
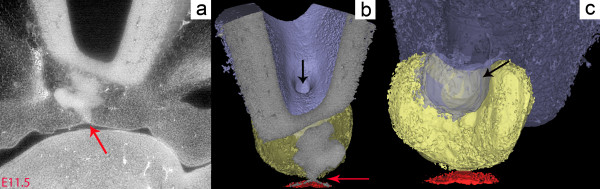
**Three-dimensional morphology of Rathke’s pouch. (a)** Rathke’s pouch at E11.5 in a wild-type mouse in coronal section, visualized using synchrotron X-ray microtomography after iodine staining. The oral ectoderm is still connected to the pouch (red arrow). **(b)** Three-dimensional reconstruction of the same sample. Anterior aspect clipped in the coronal plane. The diencephalon (blue) invaginates (black arrow) towards the pouch (yellow), which is still in continuity with the oral ectoderm (red arrow). **(c)** Posterior view of the forming pituitary at the same stage. The diencephalon evagination (black arrow) fits into a heart-shaped Rathke’s pouch (yellow) still connected to the oral ectoderm (red).

The three-dimensional (3D) morphology of the developing pituitary gland in wild-type mice at E11.5 is largely unknown and was investigated using synchrotron electron microtomography following contrast enhancement with iodine staining (Figure 2a). This novel method allows a cellular resolution to be reached in virtual sections and provides images with histological quality and sufficient contrast to allow segmentation and 3D reconstruction of small structures such as RP. At E11.5, RP is almost completely separated from the oral ectoderm, forming a heart-shaped pouch and embracing the tissue mass originating from the ventral diencephalon (Figure 2b). Interestingly, this pouch has a bilobate shape along the left-right axis (Figure 2c).

### Persistence of the buccohypophyseal canal in cilia-related mutants: a morphological study

Shh is a secreted signaling molecule required for patterning of the craniofacial midline [16,17,19,20] and RP is located in an anatomical region where Shh-related molecules are produced. We therefore focused our investigation on mutant mouse lines with known disturbances in Shh signaling.

**Figure 3:**
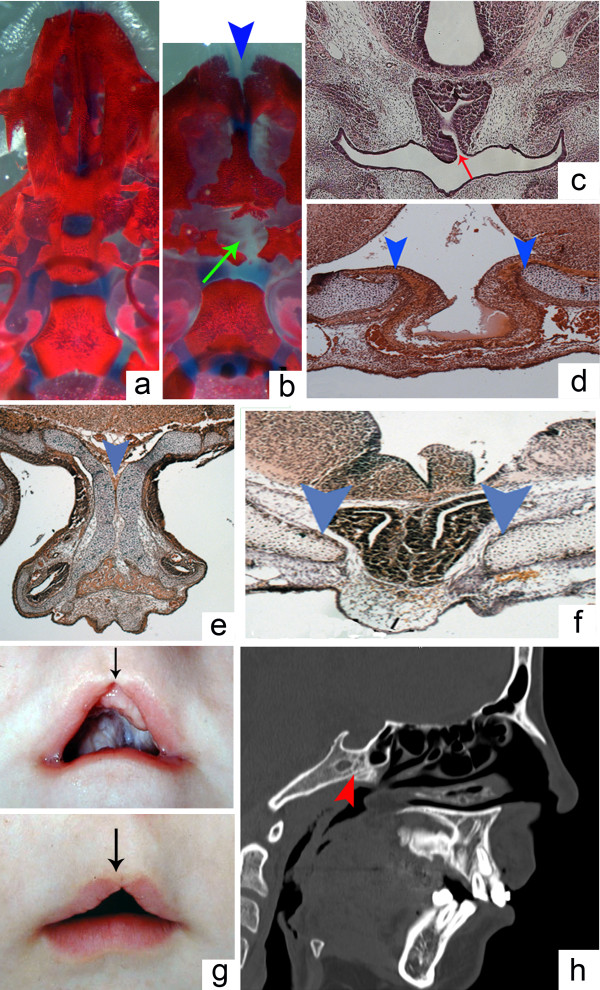
**Persistent buccohypophyseal canal and Shh pathway-related abnormalities in ciliary mutants. (a)** At E18.5, there is no midline foramen in the basisphenoid bone of wild-type mouse embryos. **(b)** In *Polaris*^*fl/fl*^*; Wnt1-Cre* mutants at E18.5, a large defect in the basisphenoid (green arrow) is associated with a midline maxillary cleft (blue arrowhead). **(c)** Histological staining showing an open buccohypophyseal canal at E18.5 in a *Polaris*^*fl/fl*^*; Wnt1-Cre* mutant (red arrow). **(d)** Basisphenoid defect in a *Kif3a*^*-/-*^ mutant at E18.5 (blue arrowheads) with bulging of the pituitary towards the oral cavity. **(e)** Duplication of the nasal septum in an *Ofd1*^*-/-*^ mutant at E18.5 (blue arrowhead). **(f)** Basisphenoid defect under the pituitary at E18.5 in an *Ofd1*^*-/-*^ mutant (blue arrowheads). **(g)** Incomplete median upper lip cleft in a patient with an *OFD1* mutation (black arrows). **(h)** Ossification defect of the sphenoid bone in the same patient (red arrowhead); standard medical computed tomography scan, mid-sagittal view at 16 years old.

Primary cilia are implicated in the regulation of Shh function [21]. Thus, to examine the possible role of Shh signaling in closure of the BHC, a phenotypic analysis of three cilia-related transgenic mice, *Polaris*^*fl/fl*^*;Wnt1-Cre*, *Ofd1*^*-/-*^ and *Kif3a*^*-/-*^, was carried out. Previous studies performed on these mice have demonstrated abnormal Shh signaling and phenotypes consistent with both reduced and increased Shh signaling activity [19,22,23]. Histological examination of *Polaris*^*fl/fl*^*;Wnt1-Cre*, *Ofd1*^*-/-*^ and *Kif3a*^*-/-*^ mice at E18.5 revealed variable malformations of the pituitary gland, commonly hindering fusion of the basisphenoid bone in the region of RP. More precisely, the pituitary gland in *Polaris*^*fl/fl*^*;Wnt1-Cre* mice was connected to the oral cavity. These mutants also had an abnormally shaped, two-headed gland, reminiscent of the anteroposterior pituitary duplications seen in *Noggin*^*-/-*^ mutants [24,25]. Examination of the skeletal preparations showed wide clefting of the basisphenoid, dysmorphic bones associated with the anterior cranial base and a generalized widening of the anterior midline structures (Figure 3a,b,c). In both *Kif3a*^*-/-*^ and *Ofd1*^*-/-*^ mutant mice, a malformed and displaced pituitary gland was noted, together with a wide opening in the basisphenoid (Figure 3d,f). In *Ofd1*^*-/-*^ mutants this was accompanied by duplications of the nasal septum (Figure 3e). Importantly, mutations of the *OFD1* gene in humans can also result in defects of the craniofacial midline. A mid-sagittal section of a medical computed tomography scan of a patient with an *OFD1* mutation (c.412G > A, p.G138S) demonstrated abnormalities of the sphenoid bone with defective ossification below the pituitary gland [26]. Clinical examination of the same patient revealed mid-facial anomalies, including a midline upper cleft lip (Figure 3g,h).

The fenestration of the basisphenoid bone and widening of the mid-facial structures are therefore common features in these cilia-related mutants. The formation of the pituitary gland is also variably disrupted in these mice and can be associated with the partial persistence of the BHC. In comparison to previous studies, these midline anomalies are close to the phenotypes associated with modifications in the expression patterns of Shh pathway-related genes [19].

### Persistence of the buccohypophyseal canal in Shh pathway-related mouse mutants: an expression pattern study

Gas1 is a glycosylphosphatidylinositol-anchored membrane protein that has been shown to act as an agonist in Shh signaling during development of the early facial midline [27,28] and as a potential antagonist in the early tooth field [29,30]. Loss of Gas1 activity results in reduced Shh signaling in the midline of the frontonasal process and in the palatal shelves. *Gas1*^*-/-*^ mice display microform holoprosencephaly, including mid face hypoplasia, cleft palate and maxillary incisor fusion [28]. Humans with mutations in *GAS1* also present with minor forms of holoprosencephaly, including maxillary hypoplasia, hypoplastic nasal septum and cleft lip or palate [31].

**Figure 4:**
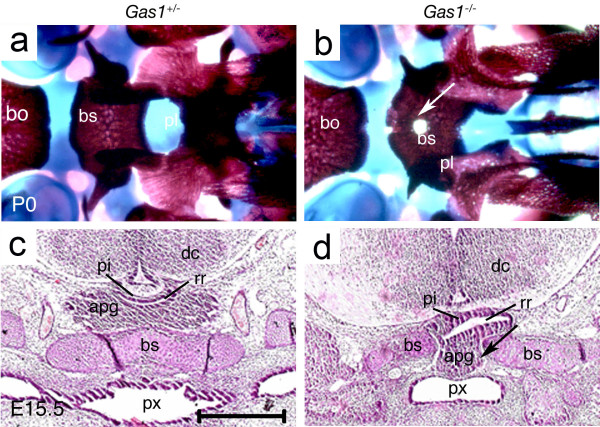
**Persistent buccohypophyseal canal in *Gas1***^***-/-***^**mutants. (a, b)** Control and mutant skeletal preparations at P0 using Alcian blue and Alizarine red showing a clearly defined bony midline defect (white arrow) in the basisphenoid. **(c, d)** Control and mutant frontal sections stained with hematoxylin-eosin showing the bony defect in the basisphenoid bone and the modification of the position of the anterior lobe of the pituitary gland in mutant (apg, black arrow**)**; the anterior lobe of the pituitary gland is located directly under the oral epithelium of the pharynx. apg, anterior lobe of the pituitary gland; bo: basioccipital bone; bs, basisphenoid bone; dc, diencephalon; pl, palatine bone; px, pharynx; pi, posterior lobe of the pituitary gland; rr, residual lumen.

Histological examination of *Gas1* mutants demonstrated a ventrally displaced pituitary gland located immediately adjacent to the oral ectoderm and interfering with closure of the basisphenoid bone, as seen also in *Polaris*^*fl/fl*^*;Wnt1-Cre*, *Ofd1*^*-/-*^ and *Kif3a*^*-/-*^ mutant mice. A ventral examination of the skeletal preparations also revealed fenestration of the basisphenoid bone (Figure 4). In order to understand the early mechanisms underlying defective pituitary gland development and basisphenoid clefting in *Gas1*^*-/-*^ mutants, we examined the expression of *Shh*, *Patched-1 (Ptch1), Gli1, Bone morphogenetic proteins-2, -4 (Bmp2, 4)* and *Fibroblast growth factors-8, -10 (Fgf8, 10)* at E10.5. The transmembrane-domain protein Ptch1 acts as the principal receptor for Shh, while Gli1 is a downstream transcription factor and transcriptional target of Shh. *Bmp2* is induced by Shh in RP where it regulates expression of different ventral transcription factors involved in cell specification of the pituitary gland [4]. Early expression of *Bmp4* from the ventral diencephalon is required for initial organ commitment and its subsequent expression in the infundibulum takes part in the development of the pituitary anlage [32]. *Fgf8* and *Fgf10* are dorsal markers expressed by the invaginating diencephalon and have an antagonistic effect on Shh. Fgf8 also has the ability to induce invagination of RP [4].

**Figure 5:**
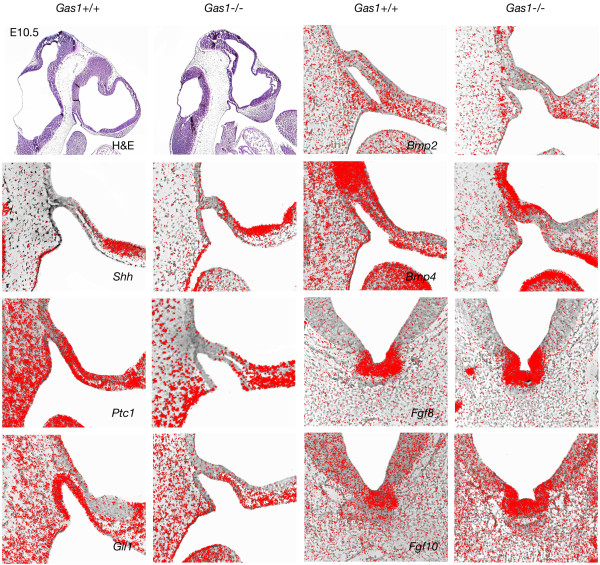
**Expression pattern of Shh pathway-related genes in *Gas1***^***-/-***^**mutants.** At E10.5, the expression patterns of *Shh*, *Bmp4*, *Bmp2*, *Fgf8* and *Fgf10* are similar in wild-type and *Gas1*^*-/-*^ mutant mice. The expression territory of *Ptch1* and *Gli1* are nevertheless reduced posteriorly and apically within Rathke’s pouch. H&E, hematoxylin and eosin.

Notably, *Shh* was similarly expressed in wild-type and *Gas1*^*-/-*^ mutant mice by the oral ectoderm posterior to RP. However, expression of the downstream factors *Ptch1* and *Gli1*, although extending throughout the ventral and dorsal RP in wild type mice, was limited to the ventral side of the anterior part of the RP in mutants. *Shh* is also strongly expressed by the ventral forebrain in close proximity to the anterior of RP, which could explain the rescued expression of *Ptch1* and *Gli1* in this region in comparison to posterior o RP. *Bmp2* and *Bmp4* expression remained unchanged in the oral ectoderm and RP in the absence of *Gas1* activity. Also, *Fgf8* and *Fgf10* were both strongly expressed in wild-type and mutant mice in the ventral invagination of the diencephalon (Figure 5). The fact that both *Ptch1* and *Gli1* were more strongly downregulated on the posterior wall of RP could also be related to the fact that the pouch is a tissue boundary between anterior neural crest-derived territories and posterior mesoderm-derived regions. In fact, we show here that a genetic disorder specifically affects gene expression at the border of neural crest and non-neural crest tissues. Although the role of this border has been emphasized in different morphogenetic processes, such as suture patency [33], differential gene expression related to this limit had not been reported. The fact that this boundary overlaps the endoderm-ectoderm boundary and the anterior limit of the notochord most probably contributes to differential gene expression on anterior and posterior sides of this limit. The altered expression of *Shh* downstream target genes in *Gas1*^*-/-*^ mutants indicates that this pathway is involved in abnormal development of the anterior pituitary gland and subsequently in persistence of foramina in the midline of the basisphenoid bone.

### A persistent buccohypophyseal canal is associated with wider midline structures in gnathostomes (jawed vertebrates)

Persistence of the BHC is considered an abnormal finding in mice and our results indicate that this is related to modifications in the Shh signaling pathway. Interestingly, the BHC is also present in a large number of fossil jawed vertebrate species as a normal anatomical feature.

In this section, we have examined the persistence of the BHC in selected chondrichthyans (cartilaginous fish) and sarcopterygians (bony lobe-finned fish) and correlate this character with key features of midline anatomy.

The purpose of this evolutionary investigation was to find morphological evidence for the involvement of Shh in BHC persistence in early vertebrates.

#### The buccohypophyseal canal and the cranial base in chondrichthyans (cartilaginous fish)

In order to investigate the evolutionary origins of BHC persistence, we first examined the cranial base of several key chondrichthyan species. These cartilaginous fish represent a vertebrate group with high variability in the anatomy of the cranial base, and we noted an interesting correlation between persistence of the BHC and the midline structure of the anterior cranial base.

**Figure 6:**
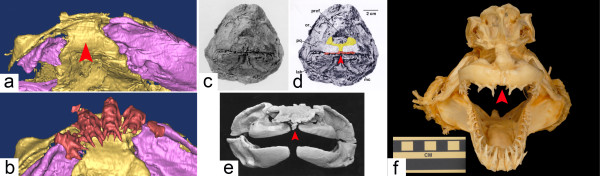
**The closure of the buccohypophyseal canal in chondrichthyans is associated with modifications of the anterior midline. (a)** Detail of the palate in *Doliodus problematicus*, NBMG 10127, Lower Devonian of Canada; three-dimensional reconstruction based on X-ray microtomography, showing palatoquadrates (in purple) widely separated by the ethmoid region (in yellow, arrowhead) (after [34]). **(b)** Same specimen with teeth (red) in place; note the presence of teeth on the ethmoid keel. **(c)** Anterior view of the head of a hybodont shark, *Egertonodus basanus* (Egerton), NHM 6356, Lower Cretaceous, Wealden, Pevensey, England (after [40]). **(d)** Same specimen, with anterior tooth positions in red, and anterior parts of the palatoquadrates colored in yellow; the distance between the palatoquadrates is narrower than in *Doliodus* (arrowhead). **(e)** Anterior view of an articulated Permian shark braincase and jaws, *Orthacanthus sp.* (probably *O. texensis*), from Archer County, Texas (cast of MCZ 12872); the palatoquadrates are still separated by a narrow band of ethmoid bone (arrowhead). **(f)** Anterior view of an articulated extant shark braincase and jaws, *Isurus oxyrinchus*, showing a fusion of the two palatoquadrates (arrowhead), only separated by the palatonasal ligament.

In many fossil chondrichthyans, such as in *Doliodus* (Figure 6a,b) [34], *Pucapampella*[35], *Orthacanthus* (Figure 6e) [36], *Cladodoides*[37], *Kawichthys*[38], *Cobelodus*[39] and *Egertonodus* (Figure 6c,d) [40], the palatoquadrates do not fuse at the midline and are separated by an ethmoidal internasal septum. In these chondrichthyan taxa, there is either a distinct, persistent BHC separated from the internal carotid canals, or a large hypophyseal fenestra - an opening in the cranial base that communicates with the hypophyseal chamber of the endocranial cavity by which the internal carotid arteries enter the neurocranium.

By contrast, we noticed that living chondrichthyans, such as the neoselachians (modern sharks and rays), and holocephalans (modern chimaeroids, ratfish), do not possess an open BHC in the adult. In neoselachians, the two palatoquadrates are fused at the symphysis in the midline, and are thus not separated by an ethmoidal internasal septum (for example, *Isurus*; Figure 6f, red arrow). The condition in holocephalans is different because the palatoquadrates are fused to the neurocranium, although they remain separated anteriorly from each other by an internasal septum [5,41]. Nevertheless, the latter is very narrow and the two nasal capsules are very close to each other in the midline.

**Figure 7:**
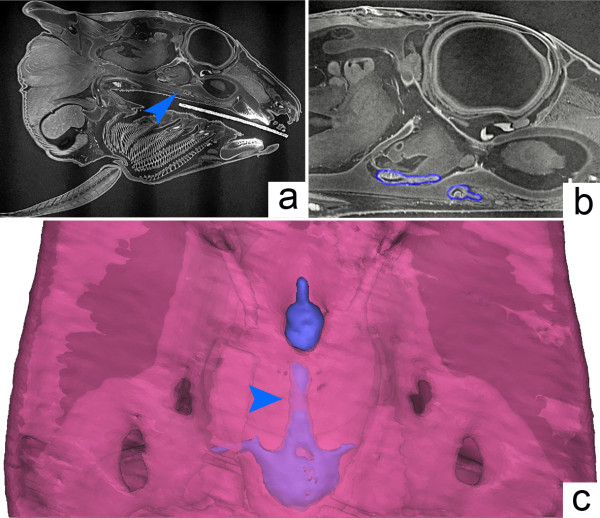
**The buccohypophyseal canal in the holocephalan *Callorhinchus*. (a)** Sagittal section obtained from synchrotron X-ray microtomography from specimen AMNH 257784, showing the pituitary region (arrowhead). **(b)** The pituitary gland has recesses on both sides of the sphenoid (outlined in blue). **(c)** Three-dimensional reconstruction in ventral view showing the pharyngeal pituitary connected to the intracranial pituitary by a long stalk (arrowhead) with zones of cartilage fusion appearing.

**Figure 8:**
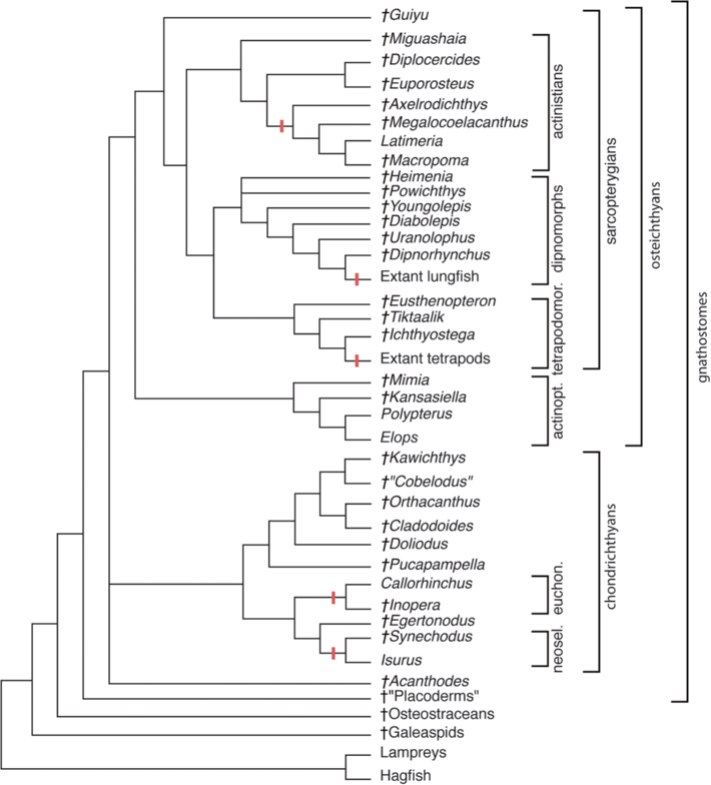
**Interrelationships of fossil and extant vertebrates.** Red lines indicate the closure of the buccohypophyseal canal. Topology based on data from literature [2,38,46,57,58]. actinopt., actinopterygians; euchon., euchondrocephalians; neosel., neoselacians; tetrapodomor., tetrapodomorphs.

Using 3D reconstructions based on synchrotron microtomography data (see Additional file 1), we show that in an extant holocephalan embryo, the BHC is partly obliterated: the cranial base separates the pituitary into two parts (Figure 7a, blue arrow and Figure 7b, blue outline) and the extracranial part aborts during ontogeny (Figure 7c, the blue arrow indicates the ongoing fusion of the cartilage in the region of the vestigial BHC). In brief, the association of a persistent BHC and wider anterior midline structures observed within basal chondrichthyans compared to living chondrichthyans (Figure 8) resemble our observations in Shh pathway-related mouse mutants.

#### The buccohypophyseal canal and the cranial base in a sarcopterygian (bony lobe-finned fish), the extant coelacanth *Latimeria chalumnae*

**Figure 9:**
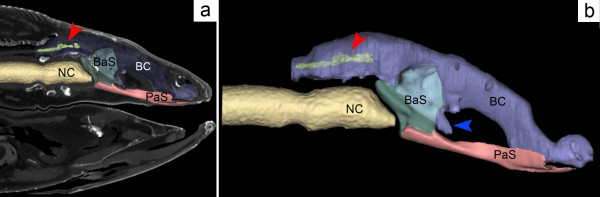
**The buccohypophyseal canal in an adult specimen of *Latimeria chalumnae.*** Three-dimensional reconstructions based on computed tomography scans of the specimen MNHN-CCC 22. **(a)** Lateral sagittal view showing the notochord (yellow), the cranial cavity (purple) containing a very small and posteriorly placed brain (red arrowhead), the basisphenoid (green) and the parasphenoid (red). **(b)** Same structures in closer view; the red arrowhead indicates the brain; the much elongated pituitary gland extends from the brain towards the skull base in a groove formed by the basisphenoid (blue arrow). BaS, basisphenoid; BC, cranial cavity; NC, notochord; PaS, parasphenoid.

Sarcopterygians are a group of osteichthyans currently comprising tetrapods, coelacanths and lungfish. Scanning of the extant coelacanth *Latimeria chalumnae* revealed that it has a partly obliterated but genuine BHC (Figure 9a and Additional file 2). In this species, the parasphenoid is densely covered with denticles on its anterior portion but does not show any foramen on its oral side. Together with the neurohypophysis, the *pars intermedia* of the pituitary gland is located ventrally to the telencephalon, whereas the *pars distalis* is situated dorsal to the roof of the oral cavity and connected to the *pars intermedia* by a very long hypophyseal duct that extends through the BHC (Figure 9b, blue arrow, and [42]). The posterior tilt of the duct is remarkable. In fact, in coelacanths, craniofacial development is most probably marked by a negative allometric growth between the brain and the endocranial cavity [43]. In pups and juveniles, the forebrain extends anteriorly to the BHC, whereas in adults the forebrain is located far posterior to these structures (Figure 9a, red arrow). Consequently, a posterior tilt of the hypophyseal duct seems to occur during postnatal development.

The braincase of basal actinistians is poorly recorded, but in the Devonian taxa in which the braincase has been described (for example, *Miguashaia*, *Euporosteus* and *Diplocercides*), the BHC always remains persistent, with an oral opening in the parasphenoid [44]. These species display a broad parasphenoid, which reaches far forward, ventrally to the nasal capsules, and have a wide interorbital septum. Furthermore, in these taxa, the brain is presumed to have extended anteriorly beyond the BHC. More derived fossil coelacanths such as the Cretaceous genera *Axelrodichthys*[45], *Macropoma*[44] and *Megalocoelacanthus*[46] display a condition similar to that of the extant genus *Latimeria* (Figure 8). Taken together, it appears that, in actinistians, the opening of the BHC into the mouth is a plesiomorphic character associated with a broad-based, platybasic braincase and further supports the possible involvement of Shh pathway-related genes in the closure of the BHC during gnathostome evolution.

## Discussion

A persistent BHC in mice results from the maintenance of the canal initiated by RP. We have first shown that RP is located at a triple tissue boundary (Figure 1) and is thus at the crossroads of several signaling pathways involved in skull base formation, including Shh. We have also shown that abnormal persistence of the BHC in mice is associated with defective Shh signaling and is correlated with anterior midline modifications. In extant and extinct vertebrate species with a normally persistent BHC in adults, we have finally found a distinctive organization of the anterior midline reminiscent of involvement of the *Shh* pathway.

Here we discuss the evolutionary importance of the position of the endoderm relative to RP. We then provide an overview of the distribution of the BHC in all vertebrate groups, discuss the current hypothesis on the functions of this canal and expose the anatomical relationships between the BHC and the carotid canals.

### Endoderm/ectoderm limit and pituitary formation

The absence of endoderm in the hypophyseal placode of mice (Figure 1) is of importance from an evolutionary point of view. It is believed that in the common jawless ancestor of all vertebrates, the hypophyseal placode and the nasal placode were fused into a single median structure called the nasohypophyseal placode [2,47]. The plesiomorphic state of the nasohypophyseal placode is thus proposed to be a single midline structure. The further steps towards the character state observed in tetrapods are considered to be: *separation* along the midline into a single midline nasal placode and a single median hypophyseal placode; and *duplication* perpendicular to the midline of the nasal placode into two paired, lateralized structures [2,48]. The duplication of the ancestral median nasal placode is believed to have been a major step towards the acquisition of jaws by vertebrates, by allowing the migration of the trigeminal neural crest cells towards the lower parts of the face [49,50]. Interestingly, in this context the heart-shaped, bilobate outline of RP at E11.5 in mice could represent an evolutionary polarity towards lateral duplication affecting the hypophyseal placode in a similar way as the nasal placode (Figure 2c).

There are only two extant jawless vertebrate groups: hagfish and lampreys. In lampreys, even though the nasal placode is a single midline structure, the development of the hypophyseal region is similar, and probably homologous to that in gnathostomes, except for dorsal shifting of the distal opening of the hypophyseal duct, due to the enlargement of the oral hood [51].

By contrast, in the case of hagfishes, there had been an ongoing controversy about whether endoderm contributes to the formation of the single midline nasohypophyseal placode, with theories based on the assumption that a posterior displacement of the endoderm boundary may have been associated with the evolutionary dislocation of the nasohypophyseal placode [52]. A recent study on hagfish ontogeny has ruled out any endodermal contribution to the hypophyseal region of both agnathan groups [53]: the absence of endoderm in the nasohypophyseal placode can now be considered as a primitive condition in vertebrates.

### Evolutionary overview of the persistence of the buccohypophyseal canal

We have already shown that, in chondrichthyans and sarcopterygians, the persistence of the BHC is associated with a broad midline. In a more general context, the evolution of the BHC raises two issues: the primitive or derived character of its maintenance and the association of its absence or presence with modifications of other midline structures.

Here we propose an overview of the different craniates groups and discuss the BHC-related evolutionary trends (Figure 8).

#### The nasohypophyseal canal in agnathans (jawless fish)

Living agnathans (cyclostomes) are the sister group of the gnathostomes. Extant groups (lampreys [2,5,54] and hagfishes [2,5,55]) as well as extinct agnathan groups, such as galeaspids [48] and osteostracans [2], possess an anteriorly open nasohypophyseal duct leading from the snout to the hypophysis, with variable pharyngeal posterior opening [2]. This duct corresponds to the invagination of a common nasohypophyseal placode [53]. The formation of this nasohypophyseal duct cannot be easily related to the formation of the BHC by persistence of RP due to its intricate ontogenetic relationships with the nasal airways [2,48,53].

#### The buccohypophyseal canal in placoderms and acanthodians (early jawed fish)

The placoderms, generally referred to as stem gnathostomes and sister group to all modern jawed vertebrates, retain an open BHC (Figure 8). This is evident in arthodires, antiarchs, phyllolepids and acanthothoracids [2,56]. The morphology of the placoderm parasphenoid varies from a flat, shallow, superficially lying denticulated plate pierced by a BHC opening, to a complicated bony element incorporated into the perichondral bone layer of the cranial base [2,56].

Among acanthodians, now considered as an extinct paraphyletic group at the base of osteichthyans (for example, *Acanthodes*, Figure 8) and chondrichthyans [57], the BHC is persistent in the few species in which the basicranium is described [2,57,58]. These data on stem gnathostomes suggest that an open BHC is a plesiomorphic condition for crown jawed vertebrates, that is, chondrichthyans and osteichthyans (Figure 8).

#### The buccohypophyseal canal in chondrichthyans (cartilaginous fish)

The fossil chondrichthyans that possess an open BHC (see Results section *The buccohypophyseal canal and the cranial base in chondrichthyans* and Figure 8) are resolved as stem chondrichthyans in recent phylogenetic studies [37,38]. By contrast, crown chondrichthyans, such as the living neoselachians and holocephalans, display a closed BHC. Moreover, basal relatives of these two modern clades (for example, the Cretaceous neoselachian *Synechodus*[59]; the Carboniferous holocephalan *Iniopera*[60]) also possess a closed canal.

The different conditions found in stem chondrichthyans and in crown chondrichthyans suggest that an open BHC is a primitive feature for chondrichthyans, and that the closure of the canal occurred in more advanced chondrichthyans.

As the closure of the canal is correlated with modifications in the midline (Figures 6 and 8), this evolutionary pattern supports the hypothesis that the transition between groups with a persistent BHC and groups with a closed BHC may have occurred by modulations in the expression territories of Shh pathway-related genes.

#### The buccohypophyseal canal in actinopterygians (bony ray-finned fish)

Within modern actinopterygians, the distribution of an open BHC does not seem to carry a strong phylogenetic signal, as the canal can be either open or closed in closely related species (for instance within the Galaxioidea [61]). Nevertheless, a persistent BHC is probably an ancestral character for actinopterygians; Paleozoic stem actinopterygians, such as *Mimia* and *Kansasiella*[62,63] possess a persistent BHC (Figure 8). *Polypterus*, which is considered as an extant example of the most basal actinopterygians, retains - to various extents - a persistent canal [2,5]. Furthermore, an open canal is retained in the teleost *Elops*, regarded as a plesiomorphic taxon of that group [64]. It is noteworthy that the development of the adenohypophysis in teleosts does not involve a RP, but instead an unfolded epithelial invagination [65], which has no major bearing on the question of the persistence of a BHC.

#### The buccohypophyseal canal in sarcopterygians (bony lobe-finned fish)

Paleozoic stem sarcopterygians such as *Guiyu*[66] have a persistent BHC. The same holds for ancestral dipnoans and coelacanths [2,44]. As seen above (see Results section *The buccohypophyseal canal and the cranial base in a sarcopterygian*), the braincases of stem actinistians and dipnoans display an open BHC with an oral opening, whereas in modern forms there is no oral opening of the BHC (Figure 9). A persistent BHC with an opening on the oral side of the parasphenoid thus appears to be the ancestral condition in sarcopterygians and actinistians (Figure 8).

The lungfishes (or dipnoans) form with the porolepiformes (a poorly diversified Paleozoic group) a clade named Dipnomorpha. LAs the coelacanths, the lungfish, like coelacanths, have a long and complex evolutionary history (more than 400 million years). Their extant forms are rare and considered as relics. The lungfishes show similar anatomical BHC-related modifications of the midline structures of the palate to the coelacanths and chondrichthyans. More precisely, in porolepiforms and early dipnomorphs such as *Powichthys*, *Youngolepis* and *Diabolepis*, the palatoquadrates are widely separated from each other by a long parasphenoid, and a BHC clearly opens in the latter. In primitive lungfish (for example, *Uranolophus*, *Dipnorhynchus*), a BHC opens in the anterior part of a short parasphenoid fused to two massive dental plates (in relation with the palatoquadrates). By contrast, crown lungfish usually show massive dental plates located close to each other along the midline and the absence of a BHC [67]. Of note, lungfish palatoquadrates are fused to the neurocranium, although this autostyly is distinct from that of holocephalans and tetrapods. Interestingly, some early porolepiforms (for example, *Heimenia*) and early dipnomorphs (for example, *Youngolepis*, *Powichthys*) show the coalescence of the grooves for the two branches of the internal carotid arteries inside a large hypophysial fossa [68,69], as seen in some fossil chondrichthyans and placoderms (see Discussion section *Relationship between the buccohypophyseal canal and the carotid canals*).

Among piscine tetrapodomorphs, both *Eusthenopteron* and *Tiktaalik* have a BHC that opens into the oral roof through the parasphenoid [2]. Within the tetrapod crown group, this character is lost, except in lissamphibians at larval stages [3]. In fact, no canal is found in any extant adult tetrapod species, but paleontological data indicate that this character state is derived: the early tetrapod *Ichthyostega*[70] had an open BHC, but the parasphenoids of younger Palaeozoic tetrapods (for example, anthracosaurs and temnospondyl labyrinthodonts) show no evidence of opening in the midline.

In summary, it appears that the BHC was open in most early vertebrates and in the stem taxa of all the major groups we examined, mainly before the Mesozoic (Figure 8). Furthermore, this overview confirms the link between the presence of the BHC and platybasic skulls and supports the potential role of Shh in the maintenance of the BHC throughout phylogeny. Interestingly, variations in the expression domains of *Shh* have previously been related to other types of phenotypic changes between related vertebrate species or between variants of a single species. In the teleost cavefish *Astyanax mexicanus*, the extension of the expression domains of Shh accounts for the evolution of eye regression [71]. Similarly, the adaptive radiation of the lower jaw in cichlid fish (teleosts), namely the difference in craniofacial anatomy between *Labeotropheus trewavasae* and *Metriaclima mbenjii*, two closely related rock-dwelling cichlid genera with very different feeding behavior, relies on modifications in expressions of genes belonging to the Shh pathway [72]. In both cases, modulations in the Shh pathway were related to eco-morphologic shifts and the phenotype change had a clear functional significance [71,72]. In the case of the BHC, the functional aspect of its maintenance is not well understood.

### Functional hypothesis on the buccohypophyseal canal

While we have provided further insight into the mechanisms leading to BHC maintenance, the function of this canal remains a mystery. Mechanosensory osmoreceptors have been described in the pituitary of the few teleosts that retain an open BHC [61,64,73] and could subsequently indicate involvement of an open BHC in osmoregulation and adaptation to the salinity of the water [74]. The persistence of this structure might suggest special environmental conditions such as permanently inconstant salinity levels in the Paleozoic basins inhabited by vertebrates [75]. Changes in the environment to the modern-type aquasphere and water salinity regime might have resulted in the obliteration of the canal independently in the evolution of various vertebrate groups. For instance, in chondrichtyans, the functions of the BHC might have been replaced by the direct osmotic regulation of urea content in blood via the epithelium of the orobranchial cavity. The canal might have become redundant after moving to other environments, such as fully marine or fully freshwater ones with more stable salinity.

Interestingly, Devonian porolepiformes and primitive lungfishes (with an open BHC) were found in marine deposits whereas almost all post-Devonian lungfishes with a closed BHC are considered as freshwater forms [76]. Furthermore, the closure of the BHC in lower tetrapods might be related to the transition from gill-breathing in water to air-breathing in amphibians. Nevertheless, the lack of an obvious environment-related distribution of species that retain an open BHC within current teleosts [61] does not provide obvious support to the osmoregulatory role of this canal.

### Relationship between the buccohypophyseal canal and the carotid canals

The BHC in placoderms is often combined with the canals for the internal carotids [77]. The occasional fusion of the BHC with the internal carotid canals results from their anatomical relationship, where they penetrate the cranial base in close proximity to each other and are bordered laterally by the paired *trabeculæ cranii*. Different types of fusions and occlusions of these three canals are observed within different groups at the time of cranial base formation [5,78].

Remarkably, within chondrichthyans, the blood supply on the cranial base follows a transition related to the anatomy of the BHC in modern groups. In neoselachians and holocephalans the dorsal and lateral dorsal aortas are free below the neurocranium, whereas in some basal chondrichthyans the dorsal aorta is enclosed in the basicranial canal (for example, *Cobelodus*[39]) and in all basal chondrichthyans the lateral dorsal aortas run into the basicranial canals (for example, *Cladodoides*[37]). In addition, in neoselachians the posterior part of the basicranial arterial system forms a bell-shaped circuit due to the laterally curving position of the lateral dorsal aortas posteriorly and the V-shaped disposition of the internal carotid arteries anteriorly [79], whereas in basal chondrichthyans the basicranial circuit is narrower, more elongated and lacks the posterior bell-shaped curve. In recent holocephalans, as well as in some primitive holocephalans (for example, *Iniopera*[60]), the internal carotid arteries abort during ontogeny [5]. Knowing that *Shh* expression is necessary for the formation of the branchial arch arteries, the transition in the structure of these arteries observed within chondrichthyans can potentially relate to a modification in *Shh* expression domains [80] and parallel the closure of the BHC.

## Conclusions

In this study we have shown that persistence of BHC in mice can arise as a result of disruptions in Shh signaling and is commonly associated with variable defects of the pituitary and the anterior midline. An open BHC appears to be an ancestral character for vertebrates [81] and, based on our evolutionary overview, we hypothesize that the opening and closure of this canal could be related to modulations in the expression of *Shh* and Shh downstream genes. Similarly, in humans with mutations in the *GLI2* gene, pituitary anomalies are reported [82] and a persistent BHC is an abnormality seen in holoprosencephaly [1]. With the current state of knowledge, the opening and/or closure of the BHC in most species seems to be a marker of variations in the dosage of *Shh* and *Shh* pathway-related genes during cranial base and midline morphogenesis.

One of the current leading theories on nasohypophyseal development is that ancestral craniates had a large median nasohypophyseal placode acting as a barrier obstructing the migration of the trigeminal neural crest on the midline and preventing the formation of jaws. The separation of the nasal placodes from the hypophyseal placode, and the subsequent formation of paired nasal placodes from a single median anterior nasal placode are considered as major events leading to the evolution of jaws, as this could have freed the anterior migration of the premandibular neural crest to form the palatoquadrates [2,47-50]. This separation was preceded by reorganization of the molecular signaling centers and Shh pathway-related proteins are good candidates for being implicated in these evolutionary transitions [83].

During ontogeny, the hypophyseal placode gives rise to a transient structure, RP, located in the anterior end of the notochord and developed in the region where three embryonic tissue boundaries meet (endoderm, neural crest-derived oral tissues and mesoderm-derived oral tissues), locating it at the crossroad of numerous signaling pathways involved in cranial base formation. After the RP separates from the oral ectoderm, it forms the anterior pituitary gland, a structure whose development is under control of Shh signaling. Our studies using mice with aberrant Shh signaling show how defective pituitary development is associated with abnormal fenestration of the basiphenoid bone and formation of the BHC, and is correlated with variable midline defects of the anterior cranial base. An evolutionary overview of chondrichthyans and sarcopterygians fish illustrates how organization of the cranial base midline structures has gone through deep remodeling and provides evidence that BHC is predominantly found as an ancestral condition within these taxa.

A single median nasal placode in the midline (holoprosencephaly [84]) and duplicated pituitaries (anterior duplication of the notochord [84]) are both described in several human syndromes, thereby confirming the fact that reorganization of these midline structures is a plausible evolutionary morphogenetic event in this region.

## Methods

### Mutant mice generation, histological analysis and skeletal preparation

*R26R-Sox17-Cre*[18], *R26R-Wnt1-Cre*[85], *Gas1*^*-/-*^[86], *Polaris*^*fl/fl*^*; Wnt1-Cre*[87], *Kif3a*^*-/-*^[88] and *Ofd1*^*-/-*^[89] mice were generated and genotyped as previously described. All animal experiments were approved by the UK Government Home Office. Timed matings were set up such that noon on the day on which vaginal plugs were detected was considered E0.5. Collection of embryonic and neonatal tissue was carried out according to the UK Home Office schedule one specification. Heads of mouse embryos were dissected, fixed in 4% paraformaldehyde in water at 4°C and dehydrated in ascending ethanol solutions before paraffin embedding. Samples were sectioned at 7 to 12 μm using a Leica semi-automated rotary microtome RM2245 (Leica Biosystems, Wetzlar, Germany). Standard hematoxylin-eosin staining was used for morphological studies. Sections were viewed in a light-field using a Zeiss microscope (Axioskope 2 plus, Zeiss, Oberkochen, Germany) and captured with an AxioCam HRC (Zeiss) using Axiovision software.

Transgenic mice carrying a LacZ reporter were processed through a LacZ staining protocol with eosin counterstaining prior to histological analysis.

For skeletal preparations, embryonic heads were de-skinned and fixed in 95% ethanol. Samples were stained for cartilage with 15 mg/ml Alcian blue 8 GX in 76% ethanol and 20% glacial acetic acid for 2 weeks at room temperature, differentiated in 95% ethanol and cleared in 1% potassium hydroxide (KOH) until bones were visible. Tissues were then stained in 0.1% aqueous Alizarin Red solution in 1% KOH and decolorized in 20% glycerol in 1% KOH. Samples were passed through a graded series of ethanol: glycerol: water solutions and stored in 100% glycerol at 4°C [90].

### Clinical data on oro-facio-digital type 1 syndrome

Written and signed consent to publish clinical pictures was obtained from the patient and her parents. The *OFD1* gene mutation was screened as previously described [26].

### Radioactive *in situ* hybridization

Radioactive *in situ* hybridization was performed according to a modified Wilkinson procedure [91]. RNA probes were labeled with ^35^S-UTP. Slides were treated with 5 μg/ml proteinase K and 0.25% acetic anhydride to reduce background. Hybridization was carried out overnight in a humidified chamber at 55°C. Slides were washed repeatedly at high stringency at 72°C and treated with 40 μg/ml RNAse A at 37°C to remove non-specifically bound probe. Sections were washed in 0.1X SSC at room temperature and dehydrated through ethanol series, air-dried and dipped in Ilford K5 emulsion. Autoradiography was performed by exposing sections in a light-tight box at 4°C for 10 to 14 days. Slides were developed using Kodak D19, fixed in Kodak UNIFIX, counterstained with hematoxylin, and mounted with DePex. For photography, dark-field images were inverted, artificially stained red, and combined with bright-field images by using Photoshop (Adobe).

### X-ray tomography protocols

The coelacanth *Latimeria chalumnae* (MNHN-CCC 20) was collected in 1960 offshore Grande Comore, Comoro Islands. The specimen is preserved is a formalin solution (7%) and was put in wet linens for computed tomography scanning using a Philips Brilliance 16 device at the Centre Hospitalier Intercommunal de Villeneuve-Saint-Georges (France). The specimen was scanned helically at a slice thickness of 800 μm, slice increment of 600 μm, 120 kV, 283 mA.

High-resolution X-ray synchrotron microtomography of mouse E11.5 embryos and of *Callorhinchus* was performed on the ID-19 beamline of the European Synchrotron Radiation Facility (Grenoble, France).

The embryo of *Callorhinchus milii* AMNH 257784 was collected from a spawning site in the Marlborough Sounds, New Zealand. The appropriate protocols for animal welfare were followed according to guidelines published by the American Society of Ichthyologists and Herpetologists. The egg capsule was collected by SCUBA diving. The egg capsule was opened within hours of collection and the embryo was removed and dissected from the yolk sac. Embryos were preserved in 10% formalin in seawater. After a minimum of one month in fixative, embryos were washed and transferred to 70% ethanol for long-term storage. This procedure followed standard protocols in place at this time (1995). Permits to collect and export the embryos were obtained from the New Zealand Ministry of Fisheries (now NIWA) and permission and support of research activities in the Marlborough Sounds was granted by the New Zealand Department of Conservation. Copies of the permits are held by the Academy of Natural Sciences in Philadelphia where the specimen was deposited. The specimen studied here is at the ontogenetic stage 36. AMNH 257784 was scanned using an isotropic voxel size of 30 μm, with a single propagation distance of 4 m. The beam was produced by the U17.6 undulator closed at the gap of 13.5 mm, altered by 2 mm of aluminum and 1 mm of copper. Such a configuration allowed us to isolate mostly a single harmonic from the original insertion device spectrum, with an effective energy of 55 keV. In order to obtain differential contrasts for the different tissues of the specimen, a single distance phase retrieval process was used [92,93]. This very simple approach allowed us to generate 3D volumes with a high sensitivity, contrast and signal-to-noise ratio, while retaining high resolution and avoiding the propagation fringes typical of edge detection scans. The full sample was scanned in six sub-scans of 4 minutes each, using 2,499 projections of 0.1 s each over 360 degrees. The detector was based on a 750-μm-thick LuAG scintillator, coupled to a fast readout low noise charge coupled device camera through a lens-based optic system. Volumes were reconstructed using ESRF software PyHST, which includes the single distance phase retrieval algorithm [92,93]. Sub-volumes were corrected for ring artifacts, and then concatenated to generate a single stack of 16 bits TIF slices.

E11.5 CD1 mouse embryos were fixed in 4% paraformaldehyde in water, dehydrated and stained using an alcoholic iodine solution [94]. The embryos were scanned using a 0.7 μm optic in binning mode (voxel size 1.356 μm). The propagation distance was 50 mm. Half-acquisition was applied in local tomography using 3000 projections of 0.2 s each. The beam was produced by the U17.6 undulator closed at the gap of 20 mm. No filter was used. The effective energy was 19.2 keV. A single distance phase retrieval process was used [76,77], coupled to an unsharp mask on the volume [77].

Imaging data were imported into the software MIMICS v.13.1 (Materialise, Leuven, Belgium) for segmentation, 3D modeling and visualization.

## Abbreviations

3D: three-dimensional; BHC: buccohypophyseal canal; E: embryonic day; KOH: potassium hydroxide; RP: Rathke’s pouch; Shh: sonic hedgehog.

## Competing interests

The authors declare that they have no competing interests.

## Authors’ contributions

RHK performed the mouse experiments, led the imaging studies and wrote the paper. MS, AO, MTC and PTS performed the mouse experiments and wrote the paper. SG, AO, AT and MR performed the mouse experiments. AP, HD, GC, OL, JGM and PJ provided and analyzed the paleontological data. CMF, BF, CJH and JH provided the mouse mutants. PT directed the synchrotron imaging study. AD provided the clinical data and performed the human mutation screening. MK did the Polaris-Wnt1-Cre knock-out and provided the histology picture of the pituitary region. All authors read and approved the final manuscript.

## Supplementary Material

Additional file 1 **Head anatomy of the holocephalan *Callorhinchus milii.*** Braincase (grey), cast of endocranial cavity and otic capsules (blue), brain and cranial nerves (green) of the late embryo of the extant holocephalan *Callorhinchus milii*, rotating from dorsal to ventral view. Three-dimensional virtual reconstruction made from synchrotron microtomography images. The oral opening of the buccohypophyseal canal is visible on the ventral aspect of the chondrocranium. See Figure 7 in the main text for further details. Click here for file

Additional file 2 **Head anatomy of the coelacanth *Latimeria chalumnae.*** Three-dimensional reconstruction of the cast of the endocranial cavity (purple), brain (green), inner ear (pink) and notochord (yellow) of the extant coelacanth *Latimeria chalumnae*, based on obtained from a standard medical computed tomography scan. See Figure 9 in the main text for further details. Click here for file
