# Correction: the buccohypophyseal canal is an ancestral vertebrate trait maintained by modulation in sonic hedgehog signaling

**DOI:** 10.1186/1741-7007-11-70

**Published:** 2013-06-21

**Authors:** Roman H Khonsari, Maisa Seppala, Alan Pradel, Hugo Dutel, Gaël Clément, Oleg Lebedev, Sarah Ghafoor, Michaela Rothova, Abigael Tucker, John G Maisey, Chen-Ming Fan, Atsushi Ohazama, Paul Tafforeau, Brunella Franco, Jill Helms, Courtney J Haycraft, Albert David, Philippe Janvier, Martyn T Cobourne, Paul T Sharpe

**Affiliations:** 1 Department of Craniofacial Development and Stem Cell Research, Comprehensive Biomedical Research Center, Dental Institute, King’s College London, London, UK; 2 Service de Chirurgie Maxillo-Faciale, Centre Hospitalier Universitaire Hôtel-Dieu, Nantes, France; 3 Department of Orthodontics, Dental Institute, King’s College London, Guy’s Hospital, London, UK; 4 American Museum of Natural History, New York, USA; 5 CNRS-UMR 7207, Muséum national d'histoire naturelle-UPMC, Paris, France; 6 CNRS-UMR 7179, Muséum national d'histoire naturelle, Paris, France; 7 Paleontological Institute of Russian Academy of Science, Moscow, Russian Federation; 8 Institute of Experimental Medicine, Academy of Sciences of the Czech Republic, Prague, Czech Republic; 9 Department of Embryology, Carnegie Institution of Washington, Baltimore, USA; 10 European Synchrotron Radiation Facility, Grenoble, France; 11 Department of Pediatrics, Università degli Studi di Napoli Federico II, Naples, Italy; 12 Department of Surgery, Stanford University, Palo Alto, USA; 13 College of Dental Medicine, Craniofacial Biology, Medical University of South Carolina, Carolina, USA; 14 Service de Génétique clinique, Centre Hospitalier Universitaire Hôtel-Dieu, Nantes, France; 15 Department of Craniofacial Development and Stem Cell Biology Dental Institute, King's College London, Guy's Hospital, London, SE1 9RT, UK

## Correction

The authors noted that the figure legend for Figure 1 (parts (a) and (b)) needs correcting [1].

The Figure 1 legend should read:

### Figure 1

**Rathke’s pouch is located at a triple boundary. a**) Whole-mount LacZ staining of an R26R-Sox17-Cre E10.5 mouse embryo showing the anterior limit of the endoderm (red arrowhead) and the posterior border of Rathke’s pouch (blue arrowhead); endobuccal view of the oral roof. (**b**) Posterior limit (arrowhead) of the neural crest-derived mesenchyme in the mid-sagittal plane at E12.5, corresponding to the location of the closing buccohypophyseal canal, in an R26R-Wnt1-Cre mouse embryo. (**c**) Anterior limit of the endoderm in the mid-sagittal plane at E10.5 (arrowhead), LacZ staining, R26RSox17- Cre mouse embryo. (**d**) Anterior end of the notochord (arrowhead), eosin staining, E10.5, wild-type mouse embryo ant, anterior; post; posterior; RP, Rathke’s pouch.

Please also note the contact address for Philippe Janvier should be: 5.CNRS-UMR 7207, Muséum national d'Histoire naturelle-UPMC, Paris, France.
